# Physiological and molecular evidence for phycobilisome degradation in maintaining carbon and nitrogen balance of cyanobacteria

**DOI:** 10.1007/s42995-025-00290-0

**Published:** 2025-04-25

**Authors:** Zhen Luo, Shuangqing Li, Muhammad Zain Ul Arifeen, Fei‑xue Fu, Huayang Gao, Taoran Sun, Lingmei Liu, Xumei Sun, Xinwei Wang, Hai-Bo Jiang

**Affiliations:** 1 School of Marine Sciences, Ningbo University, Ningbo, 315211 China; 2 Southern Marine Science and Engineering Guangdong Laboratory (Zhuhai), Zhuhai, 519080 China; 3 Southern University of Science and Technology, Shenzhen, 518000 China; 4 Department of Biological Sciences, University of Southern California, Los Angeles, 90089 USA; 5 School of Life Sciences, Central China Normal University, Wuhan, 430079 China

**Keywords:** Cyanobacteria, NblA, CCM, Gene knockout, Carbon/nitrogen balance

## Abstract

Phycobilisomes (PBS), the primary light-harvesting complexes in cyanobacteria, are degraded under nitrogen starvation to provide nitrogen for cell growth. This study reveals that carbon supply is a critical prerequisite for PBS degradation under nitrogen deficiency in *Synechococcus* sp. PCC 7002. Even under nitrogen-deficient conditions, PBS degradation is inhibited in the absence of sufficient carbon. We demonstrate that both the *nblAB*-mediated PBS-degradation pathway and the *ccmLMNK* operon-mediated CO_2_-concentrating mechanism are essential for PBS degradation. Furthermore, our findings highlight the critical role of PBS degradation in cyanobacterial adaptation to high C/N conditions. Mutant strains (Mut-*nblA* and Mut-*nblB)* deficient in PBS degradation exhibited impaired adaptation to high C/N conditions, as evidenced by their inability to thrive in high NaHCO_3_ (nitrogen-free) or CO_2_ (low-nitrogen) environments. While these mutants displayed a greener phenotype under high C/N conditions compared to the wild type, they exhibited extensive cellular damage, and significant downregulation of photosynthesis-related genes. These results provide novel insights into the carbon-dependent regulation of PBS degradation and its essential role in cyanobacterial C/N balance, highlighting its significance for their adaptation to fluctuating environmental conditions.

**Supplementary Information:**

The online version contains supplementary material available at 10.1007/s42995-025-00290-0.

## Introduction

Phycobilisomes (PBS) are essential light-harvesting complexes found in cyanobacteria, red algae, and glaucophyte algae (Domínguez-Martín et al. 2022; Zhang et al. 2017). These structures play a role in capturing light energy, transferring excitation energy to reaction centers, and regulating state transitions in photosynthetic organisms (Domínguez-Martín et al. 2022; Montgomery 2016; Takahashi and Mikami 2021; Watanabe and Ikeuchi 2013). PBS exhibit chromatic acclimation capabilities in certain cyanobacteria, adjusting their pigment composition in response to different wavelengths of light (green light, red light, etc.) to optimize light absorption and enhance the efficiency of light capture (Jiang et al. 2023; Sanfilippo et al. 2019; Takahashi and Mikami 2021; Zheng et al. 2021). PBSs are particularly large in cyanobacteria, reaching up to 5 megadalton (MDa) in molecular weight (Liu et al. 2021), making them a significant nutrient store. Consequently, their controlled degradation serves as an important strategy for cyanobacteria to cope with nitrogen deficiency. Several proteins, including NblA, NblB, NblR, NblD, and NtcA, have been implicated in PBS degradation under nitrogen deficient conditions (Hu et al. 2020; Krauspe et al. 2021; Sendersky et al. 2005, 2015). Among these, NblA is a key regulator that facilitates the interaction between Clp protease and phycobiliproteins (PBPs), leading to PBS degradation (Hu et al. 2020; Levi et al. 2018; Sendersky et al. 2015). This degradation of PBS releases amounts of nitrogen, which can then be used to support cellular growth and metabolism (Forchhammer and Schwarz 2019; Krasikov et al. 2012).

While better acclimated for nitrogen deficiency, PBS degradation in cyanobacteria is governed by a complex genetic program (Krauspe et al. 2021). NtcA, a central nitrogen deficiency regulator, is a key player in PBS degradation. It senses nitrogen status by binding the key metabolite 2-oxoglutarate (2-OG) and activates the genes involved in nitrogen uptake and assimilation (Jiang et al. 2018; Tanigawa et al. 2002; Zhang et al. 2018). In the model cyanobacterium *Synechocystis* sp. PCC 6803, NtcA directly activates the co-transcribed genes *nblA1* and *nblA2*, leading to PBS degradation (Giner-Lamia et al. 2017). Furthermore, in *Synechococcus* sp. PCC 7002 (hereafter referred to as *Synechococcus* 7002), NtcA-activated NblA expression levels increase ~ 60-fold under nitrogen deficiency (Ludwig and Bryant 2012). Upon PBS degradation, cultures change their color from blue-green to yellow and appear chlorotic (Forchhammer and Schwarz 2019). Unlike diazotrophs cyanobacteria that rely on nitrogen fixation through heterocyst cells to adapt to nitrogen deficiency, non-diazotrophs cyanobacteria rely on PBS degradation (Baier et al. 2004; Hou et al. 2015). When external nitrogen sources and internal nitrogen stores have been exhausted, non-diazotrophic cyanobacteria subsequently enter a form of dormancy (Krasikov et al. 2012).

Nitrogen and carbon metabolism are tightly linked in cyanobacteria, and a balanced ratio is required for optimal growth (Rabouille et al. 2021; Zhang et al. 2018). Nitrogen deficiency can disrupt this balance, leading to changes in both nitrogen metabolism (e.g., nitrogen uptake and assimilation, PBS degradation, heterocyst differentiation) and carbon metabolism (e.g., carbon uptake, glycogen and other carbon polymer accumulation) (Forchhammer and Selim 2020; Shimmori et al. 2018; Zeng and Zhang 2022; Zhang et al. 2018). NtcA, a key regulator in nitrogen deficiency signaling, also regulates carbon metabolism, suggesting its role in maintaining C/N balance within cyanobacteria (Herrero et al. 2004; Zhang et al. 2018). However, the precise role of NtcA-mediated PBS degradation in maintaining C/N balance in non-diazotrophic cyanobacteria under nitrogen depletion remains unclear.

Beyond nitrogen deficiency signaling, other factors appear to influence PBS degradation. The absence of nitrate/nitrite electron acceptors can lead to over-reduction of photosynthetic electron carriers, triggering a redox signal that induces *nblA* gene expression (Klotz et al. 2015). Photosynthetic activity and cell growth have also been shown to be critical for PBS degradation during nitrogen deficiency (Yoshihara and Kobayashi 2022). These observations suggest a potential role for carbon supply in regulating PBS degradation, as these factors are all indirectly dependent on carbon availability. When carbon sources are limited, cells prioritize carbon uptake and decrease nitrogen assimilation to maintain a balance with available fixed carbon (Flores et al. 2005; Forchhammer and Selim 2020). Therefore, carbon supply might play a more critical role in PBS degradation than previously recognized.

This study investigated the factors that induce PBS degradation and its role in maintaining C/N balance in the model coastal cyanobacterium *Synechococcus* 7002. By using gene knockouts, physiological analyses, and transcriptome analysis, we assessed the relative importance of nitrogen deficiency and carbon availability in regulating PBS degradation. Our findings demonstrate that both nitrogen deficiency and a sufficient carbon supply are needed for efficient PBS degradation in *Synechococcus* 7002. Furthermore, we provide evidence suggesting that the PBS degradation pathway is essential for cyanobacteria to adapt to high C/N ratio environments.

## Materials and methods

### Strains, culture conditions and general methods

*Synechococcus* 7002 was obtained from Prof. Jindong Zhao’s laboratory (Peking University, China). Cells were cultured in A^+^ medium under continuous light of 100 μmol photons m^−2^·s^−1^, at 30 ℃ with shaking at 110 rpm. The Standard A^+^ medium composition follows: 0.3 mol/L NaCl, 8 mmol/L KCl, 12 mmol/L NaNO_3_, 20 mmol/L MgSO_4_·7H_2_O, 2.5 mmol/L CaCl_2_, 8.3 mmol/L Tris–HCl (pH 8.2), 80 μmol/L Na_2_EDTA·2H_2_O, 0.37 mmol/L KH_2_PO_4_, 7.4 nmol/L cyanocobalamin (vitamin B12), and trace metals (0.55 mol/L H_3_BO_3_, 0.02 mol/L MnCl_2_·4H_2_O, 2.3 mmol/L ZnSO_4_·7H_2_O, 0.2 mmol/L Na_2_MoO_4_·2H_2_O, 12 μmol/L CuSO_4_·5H_2_O, 0.05 μmol/L CoCl_2_·6H_2_O). Kanamycin (25 μg·mL^−1^) was added to the medium for selection and maintenance of the mutant strains. All growth media, buffers were sterilized by autoclaving or filtration.

The biomass of *Synechococcus* 7002 wild-type strain and mutant strains was determined by measuring the turbidity (OD_730_) using an ultraviolet–visible spectrophotometer TU1810 (PERSEE, China) or by measuring cell numbers using a CytoFLEX flow cytometer (Beckman Coulter, America). Growth rates were calculated according to formula: ln [OD_730(Day4)_/OD_730(Day0)_]/4 or ln [cell number _(Day4)_/cell number _(Day0)_]/4 (Liu et al. 2022).

### Experimental setup

For all physiological experiments, cultures were maintained in the logarithmic growth phase for at least two generations before analysis. Cells were then harvested by centrifugation, resuspended, and washed twice with nitrogen-free A^+^ medium to remove residual extracellular dissolved nitrogen sources. Varying concentrations of NaNO_3_ were added to the medium to establish different nitrogen regimes: 0 mmol/L NaNO_3_ (nitrogen-free, N-deficient), 1.2 mmol/L NaNO_3_ (low nitrogen, low-N), and 12 mmol/L NaNO_3_ (standard nitrogen, standard-N). Different methods were employed to create carrying carbon availability. Sealed bottles were used to prevent CO_2_ entry into the medium. Additionally, the medium was sonicated for 20 min before inoculation to remove dissolved CO_2_ bubbles. For standard carbon (standard-C) conditions, gas-exchanged bottles were used, allowing CO_2_ to enter medium by shaking. To achieve high carbon (high-C) conditions, high concentrations of NaHCO_3_ were added to the medium. In low-N conditions, air or 2000 ppm CO_2_ was also bubbled into the medium to enhance carbon availability.

### Flow cytometry

Chl *a* and allophycocyanin (APC) auto-fluorescence of *Synechococcus* 7002 were measured using Cytoflex flow cytometry (Beckman Coulter, United States) with laser excitation at 488 nm (ChlA channel) and 638 nm (APC channel). Samples were diluted to a suitable concentration for the instrument's cell counting and counted 20,000 cells were analyzed for each sample. Data analysis and graphical representation of the results were performed with FlowJo 10.8.1 software (Treestar, Inc., San Carlos, CA, USA).

### Construction of mutant strains of *Synechococcus* 7002

A mutant strain with a disrupted *nblA* gene (Mut-*nblA*) was generated as an example, DNA from wild-type *Synechococcus* 7002 was used as a template to amplify the upstream segment (*nblA*-UP) and downstream segment (*nblA*-DN) flanking the *nblA* gene using specific primers (*nblA*-UP-F/R and *nblA*-DN-F/R, respectively). Recombinant plasmid P-Mut-*nblA* was obtained by inserting the *nblA*-UP fragment and *nblA*-DN fragment into the upstream and downstream of the kanamycin resistance gene (Kan^R^) of a PUC-MCS-KAN plasmid vector, respectively, and keep the gene sequences in the same orientation (Supplementary Fig. S1A). Then, the recombinant plasmid P-Mut-*nblA* was transformed into the wild-type *Synechococcus* 7002 with natural transformation method. The construction methods of the other three mutant strains (Mut-*nblB*, Mut-*ccmL-N*, Mut-*ccmK*) were consistent with the above methods. Complete segregation of the mutants was confirmed by PCR using the upstream and downstream primers (Supplementary Fig. S1). The primers, plasmids and strains used in this study are listed in Supplementary Table S2, S3 and S4, respectively.

### Pigments content and chlorophyll fluorescence

The content of Chl* a* and phycocyanin (PC), and the absorption spectrum within 600–800 nm were measured using a spectrophotometer TU1810 (PERSEE, China). Chl* a* was extracted from cells using 95% ethanol. Absorption at 648.6 and 664.1 nm of the cell extracts was then measured and Chl *a* content was calculated according to the formula: Chl *a* = 13.36 × *A*_664.1_ − 5.19 × *A*_648.6_ (Liu et al. 2022). To determine PC content, cells were collected by centrifugation, freeze-thawed repeatedly, and dissolved in PB buffer (0.1 mol/L); the absorption peaks at 438, 620 and 653 nm of protein extracts was then measured (Liu et al. 2012). PC content was calculated according to the formula: PC = [*A*_620_ − 0.195 × *A*_438_ − 0.587 × (*A*_653_ − 0.157 × *A*_438_)]/4.741. The minimal fluorescence yield (*F*_0_) of the logarithmic growth cultures was measured after 20 min of dark acclimation by using a FluorPen FP100 fluorometer (Photon System Instrument, Czech Republic).

### 2-Oxoglutarate content

Intracellular content of 2-OG was measured using the α-ketoglutarate Assay Kit (Solarbio, China). 5 × 10^6^ cells were harvested by centrifugation. Cell lysates were obtained by adding 1 mL HClO_4_ (0.3 mol/L) and ultrasonic disruption. The samples were centrifuged at 12,000 × *g* for 10 min at 4 ℃. Then, the precipitated salts (KClO_4_) in the supernatant were removed by adding 150 μL K_2_CO_3_ (2 mol/L) and centrifuging at 12,000 × *g* for 10 min at 4 ℃. L-glutamate dehydrogenase and coenzyme NADH were also quantitatively added, and the content of 2-OG in the supernatant was analyzed spectrophotometrically using glutamate dehydrogenase.

### Glycogen content

Intracellular content of Glycogen was measured using the glycogen content Assay Kit (Solarbio, China). The extraction of glycogen was accomplished through the process of alkaline hydrolysis (Vidal and Venegas-Calerón 2019), and the content of glycogen was determined by the phenol sulfuric acid method (Wang et al. 2023).

### Transmission electron microscopy

Cells from mutant strains cultured on the 4th day, and the wild-type strain cultured on both the 4th and 18th days in nitrogen-free medium were harvested by centrifugation. The cells were washed twice with phosphate buffered saline (1 mol/L), fixed with a solution containing 2.5% glutaraldehyde and 1% osmium, dehydrated with ethanol-acetone, and infiltrated with Spurr's resin. Samples were polymerized at 60 ℃ for 48 h. Ultra-thin Sects. (70 nm) were cut on an ultramicrotome-7, collected on Formvar-coated 100-mesh coper grids, and post-stained with aqueous uranyl acetate and lead citrate. Images were acquired using a transmission electron microscope Hitachi H7650 operated at 80 kV.

### RNA extraction, and transcriptome sequencing

*Synechococcus* 7002 wild-type strain and Mut-*nblA* strain were grown in standard A^+^ medium for four days, then washed by nitrogen-free A^+^ medium, and then cultured under low-N conditions with 7.7 mmol/L NaHCO_3_ addition for 48 h. About 50 mL of suspension was collected by centrifugation (5 min, 6000 rpm, 4 ℃), and frozen in liquid nitrogen. RNA was extracted using RNasey MinElute Cleanup Kit (Qiagen GmbH, Germany) and treated with RNase-free DNase I. Subsequently, a cDNA library for *Synechococcus* 7002 was constructed. Briefly, mRNA was enriched by an rRNA removal kit, fragmented, and used to synthesize cDNA. Raw data were obtained by Illumina Novaseq 6000. For gene transcript level statistics, genes with the parameter of |log2 Fold Change (FC)| ≥ 1 and adjusted *P* value < 0.05 were considered to differ in gene expression (i.e., differently expressed genes: DEGs).

### Intracellular C/N ratio

To measure intracellular C/N ratio, 10 mL cultures from each treatment were filtered onto GF/F glass microfiber filters (Whatman, 25 mm) and then washed twice with pure water and dried at 60 ℃ to constant weight. All the containers used to collect samples were pre-treated at 500 ℃ for 5 h to remove contaminating inorganic carbon. The mass percentage of C and N in the sample was measured by FlashSmart elemental analyzer (Thermo, America). The specific methods are as follows: (1) the aspartic acid standard was weighed according to a weight gradient and burned at 1150 ℃ by an elemental analyzer; (2) the C and N elements in the standard were converted to CO_2_ and N_2_, which were detected by the instrument to prepare the calibration curve; and (3) the mass percentages of C and N in the sample were converted by the calibration curve, and the C/N ratio was determined.

### Data statistics and analysis

All data have been presented as the mean and standard deviation biological replicates (*n* = 3), and independent sample t tests were used to assess differences, which have been illustrated on figures with different letters (*P* < 0.05).

## Results

### Physiological response and C/N ratio of *Synechococcus* 7002 during N-deficient adaptation

**Fig. 1:**
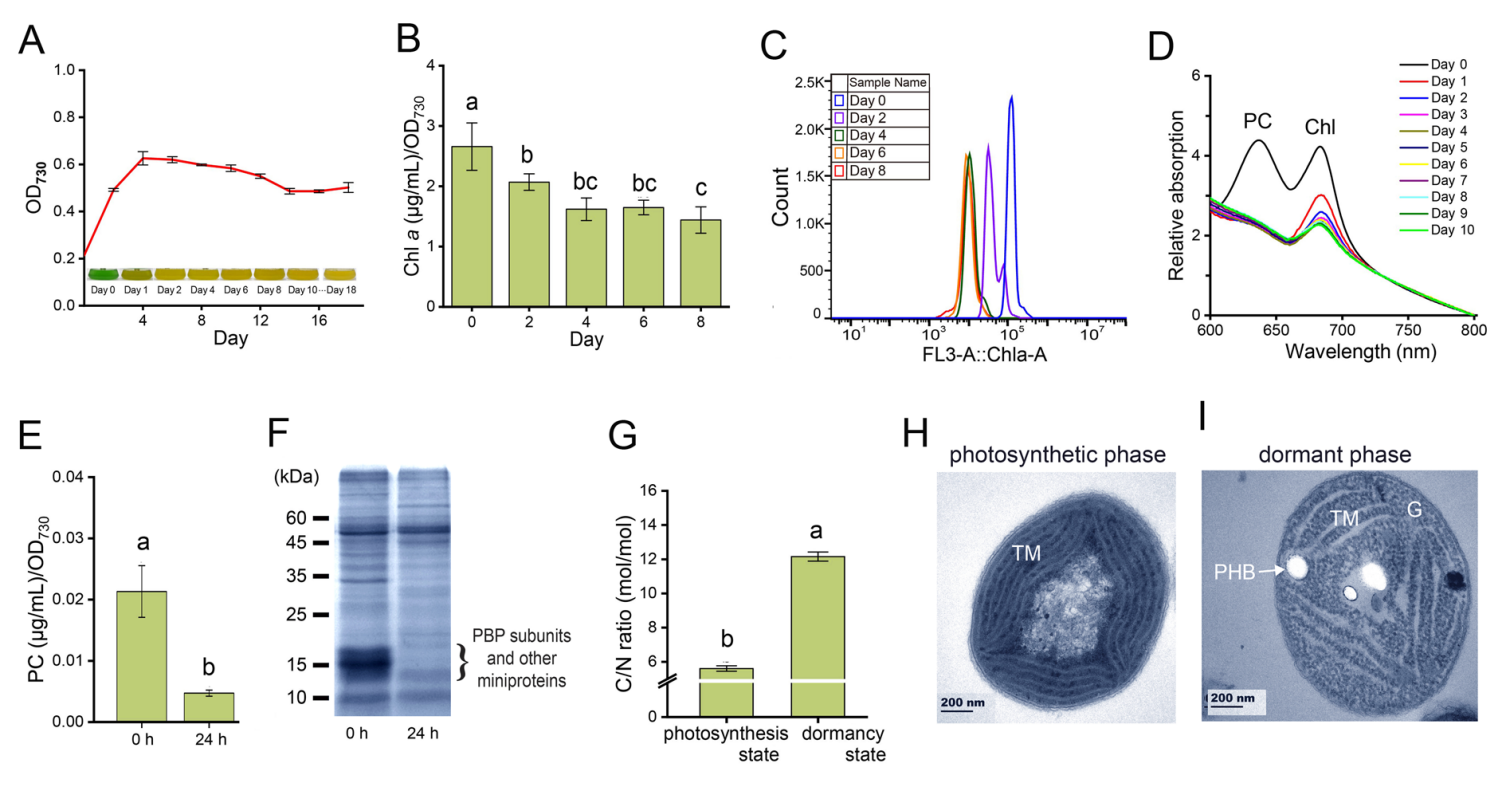
Physiological phenotypes of *Synechococcus* 7002 during long-term N-deficient incubation. **A** Growth curve and photographs of *Synechococcus* 7002 under N-deficient conditions for 18 days. **B** Chl *a* content of *Synechococcus* 7002 under N-deficient conditions. **C** Fluorescence histogram of *Synechococcus* 7002 under N-deficient conditions. The x-axis is Chl *a* auto-fluorescence of cells, the y-axis is cell numbers. **D** The absorption spectrum of *Synechococcus* 7002 within 600–800 nm under N-deficient conditions. **E** PC content of *Synechococcus* 7002 under N-deficient conditions. **F** SDS-PAGE gel of total proteins in *Synechococcus* 7002 under N-deficient conditions. **G** C/N ratio of *Synechococcus* 7002 under N-deficient conditions on the photosynthesis phase (Day 0) and dormant phase (Day 18). **H**, **I** Transmission electron microscopy pictures of the *Synechococcus* 7002 cells under N-deficient conditions on photosynthesis phase (Day 0) (**H**) and dormant phase (Day 18) (**I**). *TM*, thylakoid membranes; *G*, glycogen granules; *PHB*, polyhydroxybutyrate granules. The error bars in the figures represent standard deviation between three replicates, and observed differences are marked with lowercase letters on each bar, with different letters representing significant differences, (*P* < 0.05)

When cultured in N-deficient conditions for 18 days, *Synechococcus* 7002 cultures exhibited chlorosis, with a color change from blue-green to yellow after 24 h (Fig. 1A). Chlorophyll *a* (Chl *a*) content significantly decreased during the initial four days (Fig. 1B), accompanied by a leftward shift in Chl *a* peaks, observed by flow cytometry (Fig. 1C), indicating Chl *a* degradation or an inhibited Chl *a* biosynthesis within four days. Phycocyanin (PC), a major PBS component in cyanobacteria, contributed a distinctive absorption peak at 620 nm. This peak disappeared after one day of N-deficiency, and PC content significantly decreased (Fig. 1D, E), suggesting PC degradation. Additionally, bands around 15 kDa in SDS-PAGE gel, corresponding to phycobiliprotein (PBP) subunits, almost completely disappeared after 24 h (Fig. 1F). These results collectively suggest PBS degradation in *Synechococcus* 7002 under N-deficient conditions, likely contributing to its nitrogen acquisition strategy. Furthermore, C/N ratios and submicroscopic structure were investigated in *Synechococcus* 7002 under N-deficient conditions. The intracellular C/N ratio of cells cultured on the 18th day of N-deficiency was 2.7-fold higher than that of cells in initial photosynthetic state (Day 0) (Fig. 1G). This suggests a shift in cellular resource allocation under N-deficiency. Consistent with this, transmission electron microscopy (TEM) revealed the presence of polyhydroxybutyrate (PHB) granules in cells grown under N-deficiency for 18 days, but not in initial photosynthetic state (Day 0) (Fig. 1H, I). The accumulation of PHB, a carbon storage compound, suggests the conversion of excess carbon into storage molecules in response to nitrogen depletion, contributing to the elevated C/N ratio.

### Carbon availability regulates PBS degradation in cyanobacteria under nitrogen deficiency

**Fig. 2:**
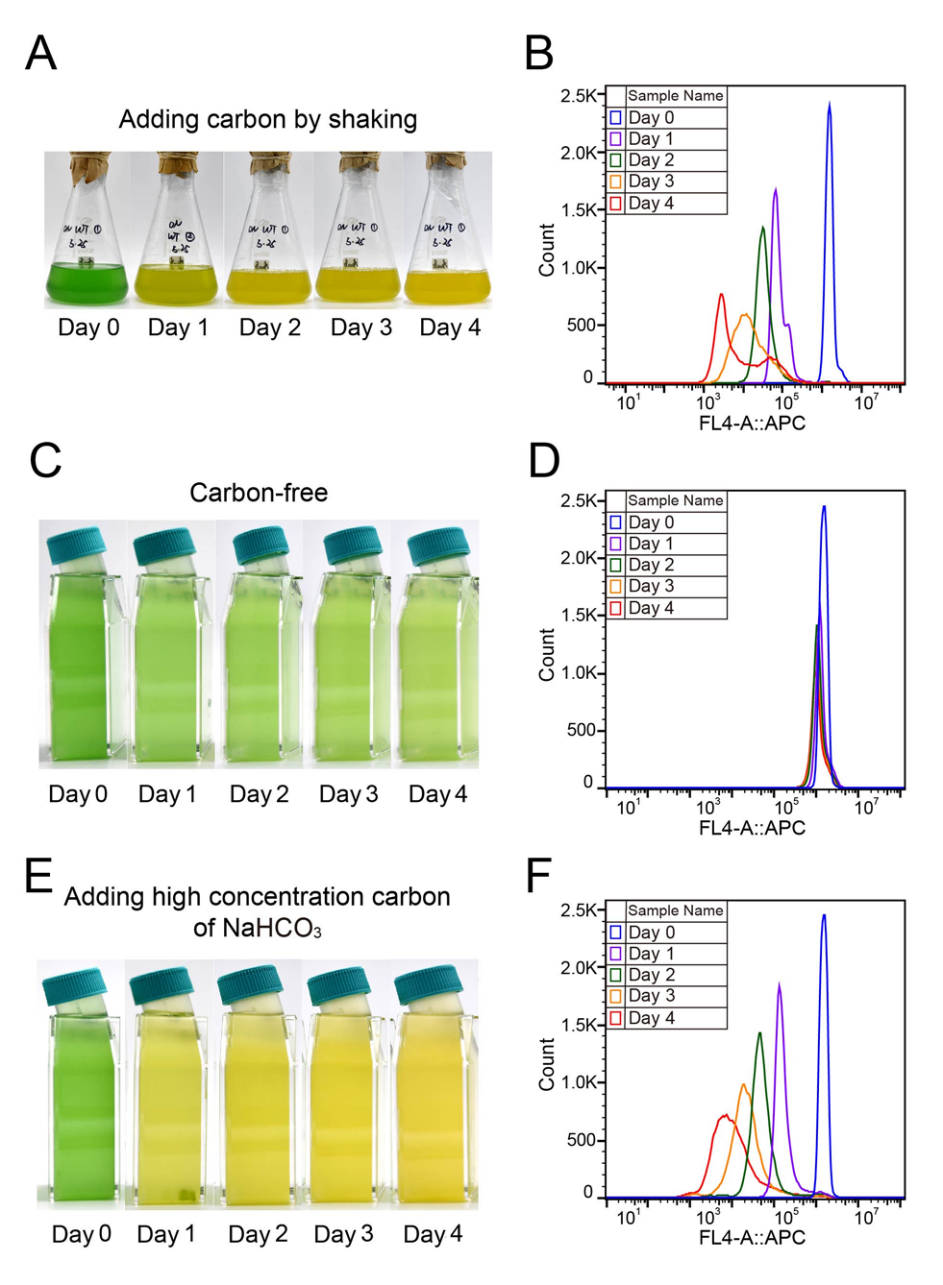
Photographs and flow cytometry of N-deficient *Synechococcus* 7002 cultures when supplied with different carbon sources. **A**, **B** Photographs (**A**) and fluorescence histogram (**B**) of N-deficient *Synechococcus* 7002 cells cultured in gas-exchanged bottles that CO_2_ can enter culture medium by shaking (standard-C). **C**, **D** Photographs (**C**) and fluorescence histogram (**D**) of N-deficient *Synechococcus* 7002 cells cultured in sealed bottles that CO_2_ cannot enter culture medium (carbon-free). **E**, **F** Photographs (**E**) and fluorescence histogram (**F**) of N-deficient *Synechococcus* 7002 cells cultured in sealed bottles but added 7.7 mmol/L NaHCO_3_ (high-C). The x-axis is APC auto-fluorescence of cells, the y-axis is cell numbers

To investigate the role of carbon supply in cyanobacterial PBS degradation under nitrogen deficiency, *Synechococcus* 7002 cells were cultured in sealed bottles with no addition of carbon source, but supplemented with high concentration of NaHCO_3_ and compared it with gas-exchanged cultures. Unlike gas-exchanged cultures where PBS degradation occurred readily after one day of cultivation cells cultured in sealed bottles remained green for > 4 days and exhibited no PBS degradation (Fig. 2C). Flow cytometry supported this observation, showing minimal leftward shift in the APC auto-fluorescence peak in N-deficient cells cultured in sealed bottles (Fig. 2D), compared to the significant shift observed in in gas-exchange cultures (Fig. 2B). These results suggest that *Synechococcus* 7002 cannot degrade PBS under N-deficient conditions without sufficient carbon supply. However, when a high concentration of NaHCO_3_ (7.7 mmol/L) was added to the sealed cultures, rapid PBS degradation was observed within a day of exponential growth (Fig. 2E, F). These results further support the importance of carbon supply for PBS degradation in N-deficient cyanobacteria.

### Requirement of Nbl-system and CO_2_ concentrating mechanism for PBS degradation

**Fig. 3:**
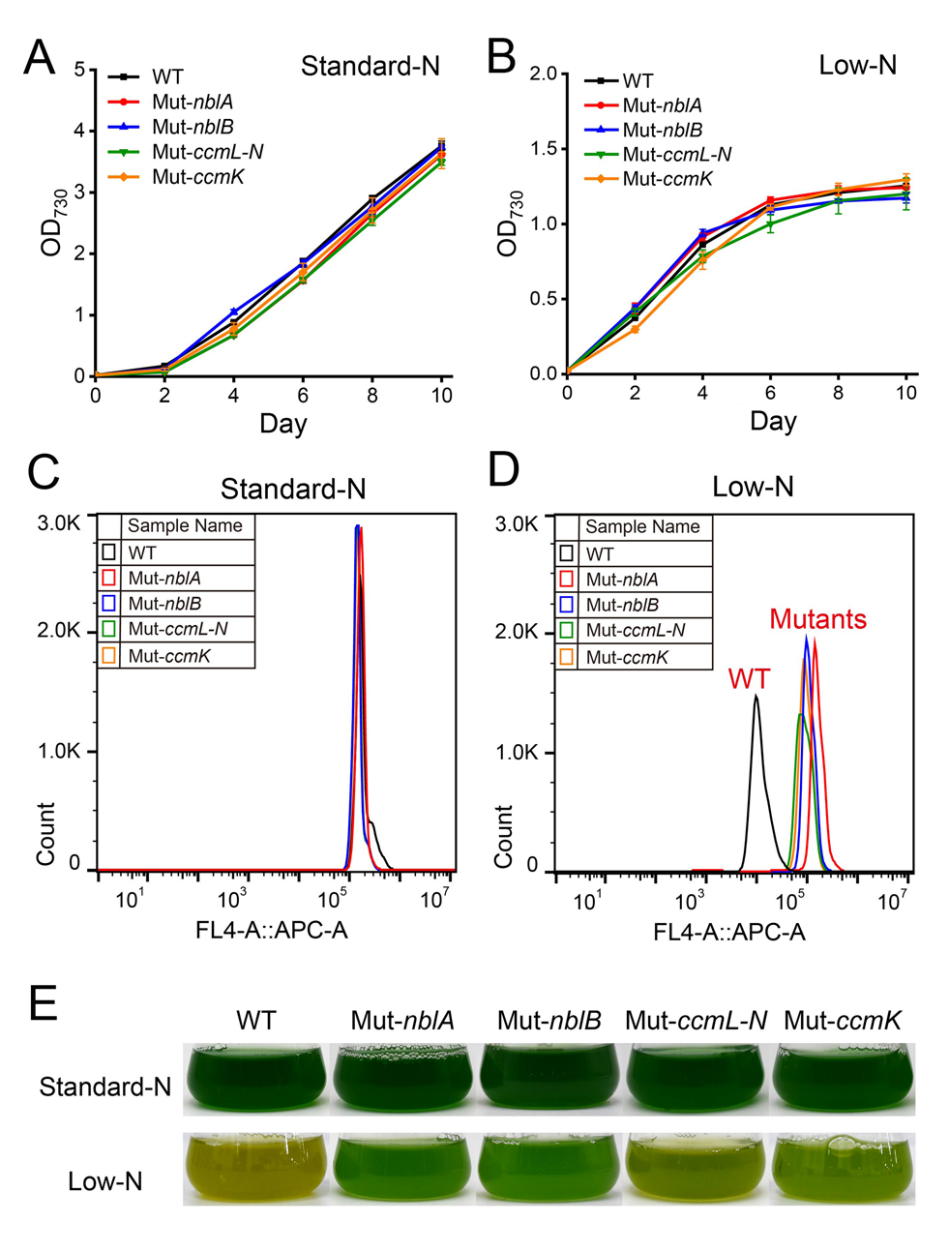
Growth curves, APC auto-fluorescence changes and photographs of *Synechococcus* 7002 wild-type strain and mutant strains under standard-N (12 mmol/L NaNO_**3**_) and low-N (1.2 mmol/L NaNO_**3**_) conditions. **A**, **B** Growth curves of *Synechococcus* 7002 wild-type strain and mutant strains under standard-N (**A**) and low-N (**B**) conditions. **C**, **D** Fluorescence histogram of *Synechococcus* 7002 wild-type strain and mutant strains on the 4th day under standard-N (**C**) and low-N (**D**) conditions. The *x*-axis is APC auto-fluorescence of cells, the *y*-axis is cell numbers. **E** Photographs of the strains on the 4th day under standard-N and low-N conditions

To obtain *Synechococcus* 7002 strains incapable of PBS degradation, we constructed two mutant strains (Mut-*nblA* and Mut-*nblB*) by knocking out *nblA* and *nblB* genes (detailed in Experimental procedures, Supplementary Fig. S1). PCR analysis supported successful knockout of the target genes in the mutant strains. When cultured in gas-exchanged bottles, these mutants exhibited growth similar to the wild-type under both standard-N (12 mmol/L) and low-N (1.2 mmol/L) conditions (Fig. 3A, B). The inactivation of *nblA* and *nblB* had no influence on color and APC auto-fluorescence under standard-N conditions (Fig. 3C, E). However, unlike the wild-type, Mut-*nblA* and Mut-*nblB* strains did not degrade PBS when cultured under low-N conditions. The peak of APC auto-fluorescence in the mutants remained unchanged, and the mutants did not occur chlorosis like the wild-type strain (Fig. 3D, E).

Cyanobacteria rely on CO_2_ concentrating mechanism (CCM) to uptake and transport CO_2_ from the environment into cells. To further explore the effect of carbon availability on PBS degradation in cyanobacteria, CO_2_ concentrating mechanism proteins, *ccmK* and *ccmL-N* were knocked out (for detail see the Experimental procedures section). Similar to Nbl-system mutants, inactivation of CCM-related genes also had significant effects on PBS degradation of *Synechococcus* 7002. When cultured in gas-exchanged bottles, the CCM-deficient mutants were unable to effectively degrade PBSs under low-N conditions (Fig. 3D, E). These findings suggest that both carbon supply, facilitated by CCM, and the Nbl-system are essential for cyanobacterial PBS degradation under nitrogen deficiency.

### Defective PBS degradation impairs adaptation to high C/N stress conditions

To explore the significance of PBS degradation for cyanobacterial cells under high C/N conditions, wild-type and PBS-degradation deficient mutants (Mut-*nblA* and Mut-*nblB*) were cultured in three different high C/N ratio conditions.

1. **High C/N with NaHCO**_**3**_** in gas-exchanged bottles**

**Fig. 4:**
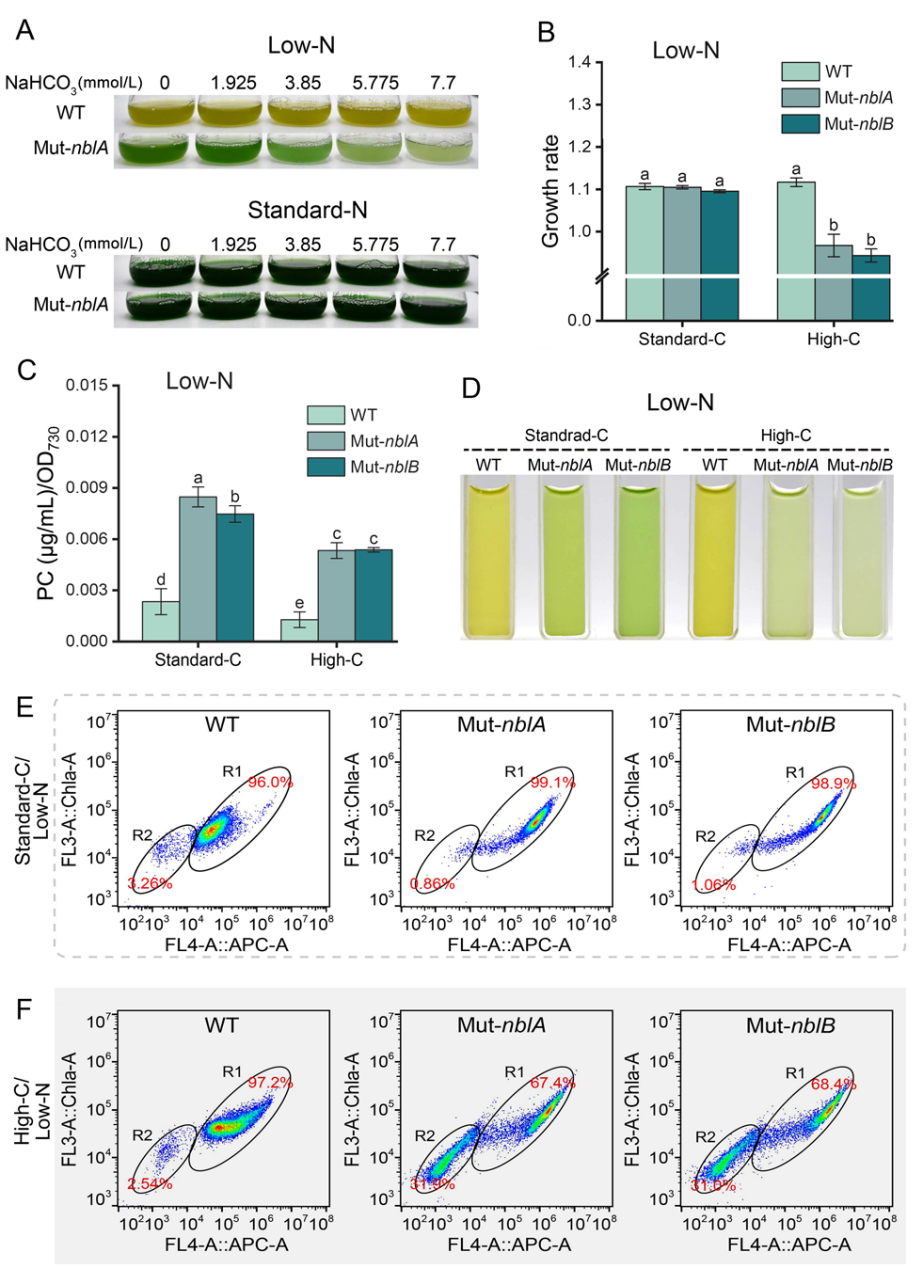
Physiological characteristics of *Synechococcus* 7002 wild-type strain, Mut-*nblA* and Mut-*nblB* strains under different culture conditions. **A** Photographs of the wild-type strain and Mut-*nblA* strain on the 4th day under standard-N (12 mmol/L NaNO_3_) and low-N (1.2 mmol/L NaNO_3_) conditions with NaHCO_3_ addition. **B**–**D** Growth rate (**B**), PC content (**C**) and photographs (**D**) of wild-type strain, Mut-*nblA* and Mut-*nblB* strains under low-N and low-N with 7.7 mmol/L NaHCO_3_ addition (high-C) conditions. **E**, **F** Flow cytometry scatterplots of the wild-type strain, Mut-*nblA* and Mut-*nblB* strains under standard-C (CO_2_ enter medium by shaking) (**E**) and high-C (**F**) conditions, all these conditions are under low-N medium containing 1.2 mmol/L NaNO_3_. The x-axis is APC auto-fluorescence of cells, the y-axis is Chl *a* auto-fluorescence of cells. The error bars in the figures represent one standard deviation, and significance analysis is marked with lowercase letters on each bar, with different letters representing significant differences, (*P* < 0.05)

Under standard-N conditions with varying NaHCO_3_ concentration, no significant differences in color between the PBS-degradation deficient mutants and wild-type strain were observed, and all strains remained green (Fig. 4A). This suggests that adding a carbon source does not affect PBS degradation under standard-N conditions. However, under low-N (1.2 mmol/L) conditions with 7.7 mmol/L NaHCO_3_, the growth of the mutants was significantly inhibited compared to that of the wild-type strain (Fig. 4A, B, D). The mutants maintained higher PC content (Fig. 4C) but had a significantly lower growth rate than that of the wild-type strain (Fig. 4B). Flow cytometry showed ~ 30% of the mutant cells formed low Chl *a* and low APC groups after NaHCO_3_ addition (Fig. 4E, F). These results imply that PBS degradation mechanisms facilitate cyanobacterial adaptation to high C/N ratio environments.

(2) **High C/N with NaHCO**_**3**_** in sealed bottles**

**Fig. 5:**
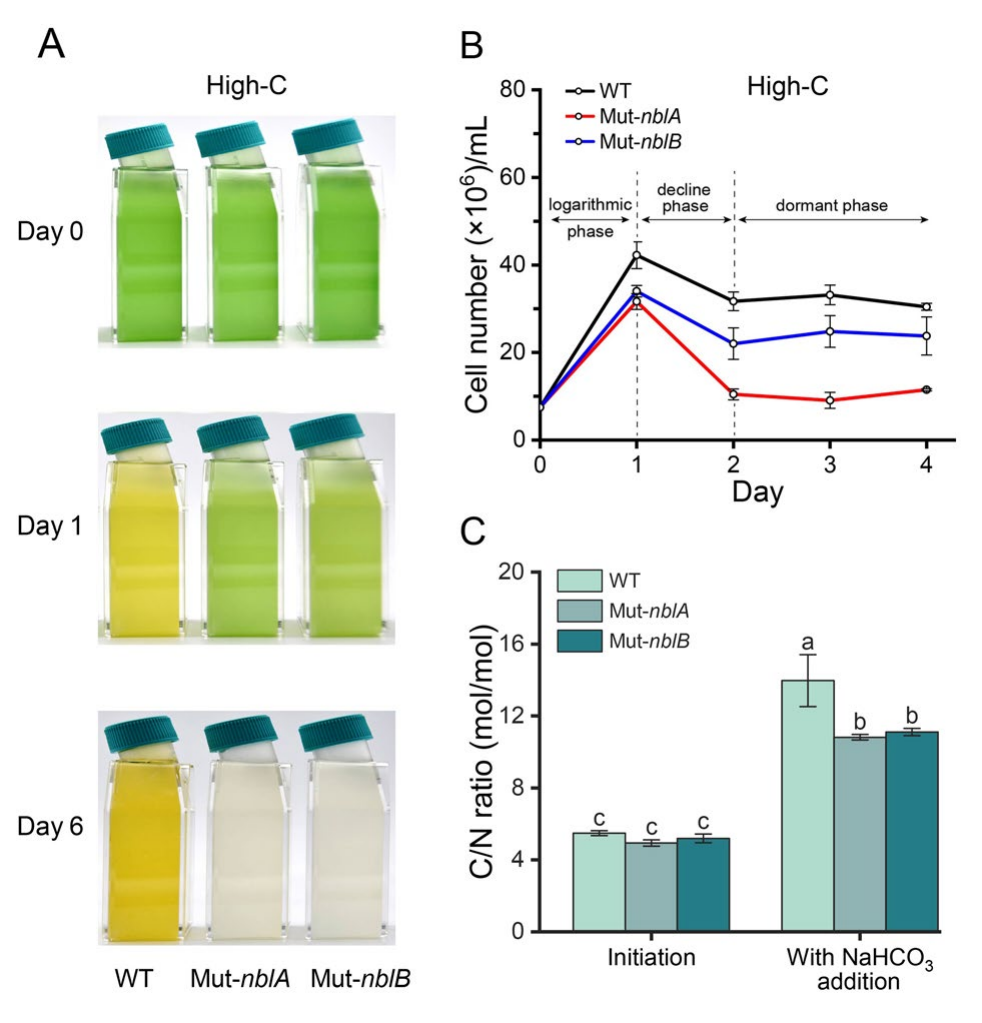
Photographs (**A**), growth curves (**B**) and C/N ratio (**C**) of N-deficient *Synechococcus* 7002 wild-type strain, Mut-*nblA* and Mut-*nblB* strains under high-C (with 7.7 mmol/L NaHCO_3_ addition) conditions. The error bars in the figures represent standard deviation between three replicates, and significance analysis is marked with lowercase letters on each bar, with different letters representing significant differences, (*P* < 0.05)

When cultured in sealed bottles under N-deficient conditions with 7.7 mmol/L NaHCO_3_, the wild-type strain rapidly underwent chlorosis, grew slowly, and soon entered a dormant phase (Fig. 5A, B). Though the PBS-degradation deficient mutants did not exhibit chlorosis, growth was significantly inhibited compared to the wild-type strain (Fig. 5A, B), and cultures declined by the 6th day (Fig. 5A). The wild-type strain maintained a high C/N ratio during dormant phase (Day 4), while the mutants had a significantly lower C/N ratio (Fig. 5C). These results suggest that PBS degradation plays a role in maintaining intracellular C/N balance. The lack of PBS degradation disrupted the carbon and nitrogen balance in the mutants, leading to cell death under high C/N stress.

(3) **High carbon with high CO**_**2**_** in gas-exchanged bottles**

**Fig. 6:**
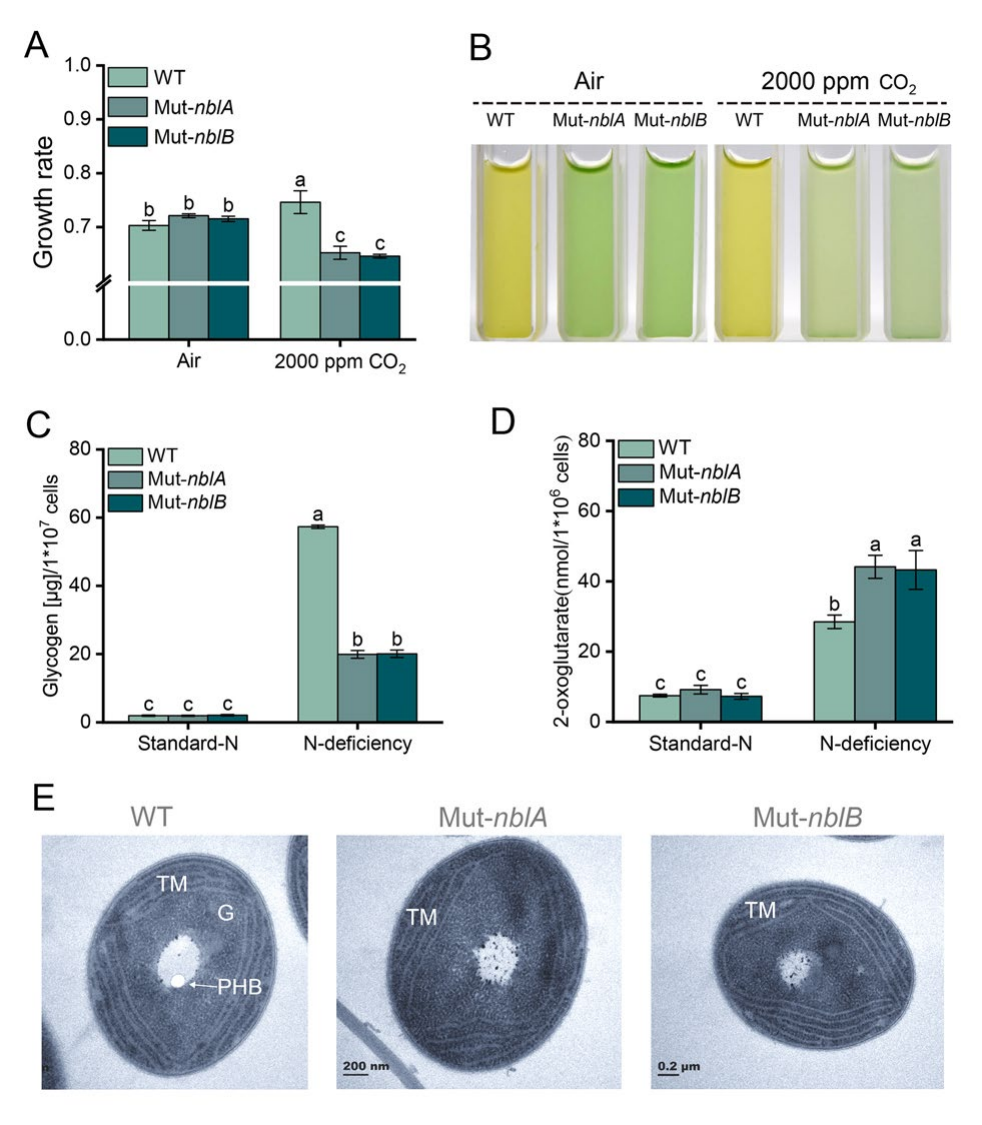
Growth rate, photographs and changes of intracellular matter of *Synechococcus* 7002 wild-type strain, Mut-*nblA* and Mut-*nblB* strains. **A**–**B** The growth rate (**A**) and photographs (**B**) of the strains on the 4th day under low-N with air or 2000 ppm CO_2_ conditions for four days. **C** The intracellular content of glycogen of the strains on the 4th day under standard-N and N-deficiency conditions with standard-C. **D** The intracellular content of 2-OG of the strains on the 2nd day under standard-N and N-deficiency conditions with standard-C. **E** TEM images of the strains under N-deficient conditions with standard-C. *TM*, thylakoid membranes; *G*, glycogen granules; *PHB*, polyhydroxybutyrate granules. The error bars in the figures represent standard deviation between three replicates, and significance analysis is marked with lowercase letters on each bar, with different letters representing significant differences, (*P* < 0.05)

When exposed to high CO_2_ concentration (2000 ppm) under low-N conditions, similar results of high C/N sensitivity were observed. The growth of the PBS-degradation deficient mutants was significantly inhibited compared to the wild-type strain, with a growth rate decrease of ~ 10% over the 4th day incubation (Fig. 6A, B). Flow cytometry revealed that ~ 30% of the mutant cells formed low Chl *a* and low APC groups when exposed to high CO_2_ concentration (Supplementary Fig. S2A, B), consistent with previous findings (Fig. 4). Intracellular glycogen content and TEM images revealed significant differences between the wild-type strain and the PBS-degradation deficient mutants when cultured in N-deficient medium with standard-C for four days. The mutants exhibited significantly lower glycogen content compared to the wild-type strain (Fig. 6C, E). Additionally, PHB particles were not observed in the mutants (Fig. 6E). Furthermore, the intracellular 2-OG content in mutants was ~ 1.5 times higher than that of the wild-type strain on the 2nd day under N-deficiency conditions with standard-C, indicating that the stress of nitrogen deficiency was more severe (Fig. 6D). These results indicate that the PBS-degradation deficient mutants not only lost the ability to regulate intracellular carbon flow but are also subjected to a more severe nitrogen deficiency stress, which affects their ability to maintain C/N balance.

### PBS-degradation deficiency disrupts photosynthesis-related genes under high C/N stress

**Fig. 7:**
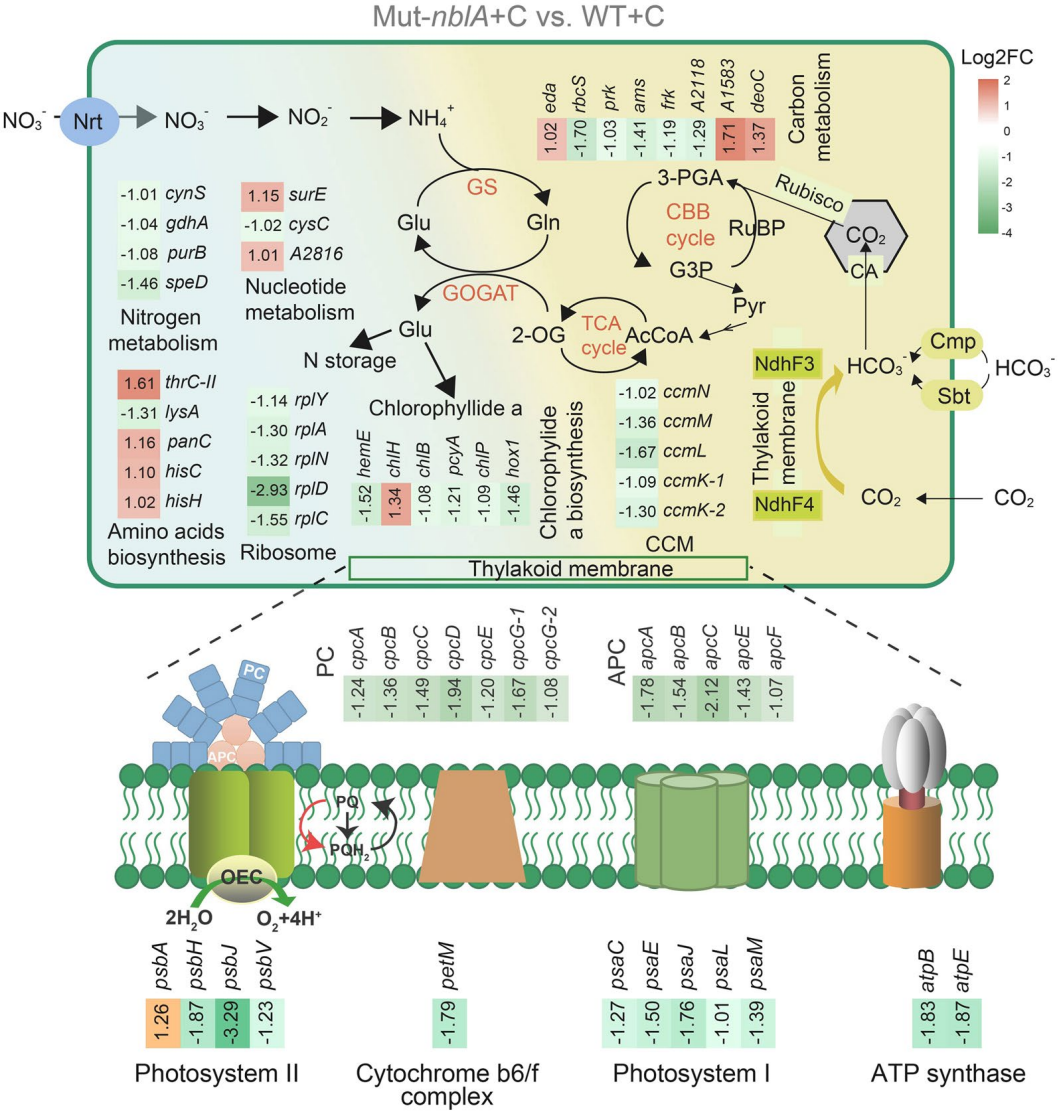
Transcriptome analysis of the regulatory network of carbon and nitrogen metabolism of the Mut-*nblA* strain under high C/N conditions in *Synechococcus* 7002. Mut-*nblA* strain compared to wild-type strain DEGs in different metabolic pathways are shown using heat maps with gene names or accession IDs. *Nrt*: ABC-type nitrate/nitrite transporter; *Cmp*, *Sbt*: two high affinity ATP-dependent ABC-type HCO_3_^−^ transporters; *CA*: carbonic anhydrase; *2-OG*: 2-oxoglutarate; *TCA cycle*: tricarboxylic acid cycle; *AcCoA*: acetyl-CoA; *Pyr*: pyruvate; *G3P*: glucose-3-phosphate; *3-PGA*: 3-phosphoglycerate; *CBB cycle*: calvin-benson-bassham cycle; *RuBP*: 1, 5-ribulose bisphosphate; *CCM*: CO_2_ concentrating mechanism; *APC*: allophycocyanin; *PC*: phycocyanin

RNA-sequencing analysis revealed significant differential expression in over 12% of Mut-*nblA* genes compared to the wild-type under high C/N conditions. Notably, 211 genes were downregulated, while 146 were upregulated in the mutant (Supplementary Fig. S3A). KEGG enrichment analysis identified a significant downregulation of genes involved in nitrogen utilization pathways (e.g., nitrogen metabolism, chlorophyllide *a* biosynthesis, and ribosome biosynthesis), photosynthetic apparatus assembly pathway, and PBS assembly pathway under high C/N conditions (Fig. 7). Upregulated genes were mainly involved in nucleotide metabolism and amino acid biosynthesis (Supplementary Fig. S3B). Among the genes related to carbon metabolism, most genes were downregulated in the mutant strain, especially those related to CCM. Only a few genes in this pathway showed upregulation (Fig. 7). For a summary of theses significantly expressed genes see Supplementary Table S1. These findings suggest that the loss of PBS-degradation machinery adversely affect both carbon and nitrogen metabolism in cyanobacteria under high C/N ratio environments.

## Discussion

Cyanobacteria degrade PBS during nitrogen deficiency for two main reasons: (1) to reduce light-harvesting antennas, preventing excessive reduction of photosynthetic electron carriers and potential photo-damage (Salomon et al. 2013) and (2) to recycle nitrogen from PBS proteins to support other cellular functions, maintaining growth and viability under nitrogen-limited conditions (Ruan et al. 2018). Our findings revealed that nitrogen deficiency alone is not sufficient for PBS degradation but also requires carbon supply. The relative ratio of bioavailable carbon and nitrogen, needed for cell growth and metabolic activities like photosynthesis, appears to be the determinant of PBS degradation. *Synechococcus* 7002 degraded PBS under nitrogen limitation when there was a sufficient external carbon supply. Cultures grown in sealed bottles without CO_2_ influx (limited carbon) did not exhibit PBS degradation despite N-deficiency (Fig. 2). Similarly, mutants with knocked out CCM-associated proteins (Mut-*ccmN-L* and Mut-*ccmK*), which reduce CO_2_ uptake and fixation, showed little to no PBS degradation under low-N conditions (Fig. 3). However, when there is a sufficient carbon supply under low-N or N-deficient conditions, whether through shaking, bubbling CO_2_, or directly adding high concentrations of NaHCO_3_, PBS degradation occurred (Figs. 4, 5 and 6). These observations reveal the importance of sufficient carbon uptake for triggering PBS degradation.

Similar observations have been made during heterocyst differentiation in *Anabaena* sp. PCC 7120 (Gollan et al. 2020). Traditionally, nitrogen deficiency has been considered the sole trigger for heterocyst differentiation. However, these studies show that even in the presence of combined nitrogen sources, *Anabaena* can differentiate heterocysts to increase nitrogen uptake when exposed to high CO_2_ concentrations (Gollan et al. 2020; Zeng and Zhang 2022). These findings suggest that C/N imbalance, rather than just nitrogen deficiency, may be the driver of heterocyst differentiation (Herrero and Flores 2019; Zeng and Zhang 2022). Similar to heterocyst differentiation, our work highlights that PBS degradation is primarily induced by a disrupted C/N balance, not solely by nitrogen deficiency. Both of these processes are important physiological processes to cope with nitrogen deficiency. In this study, we proposed that not only nitrogen deficiency but also carbon supply induces these physiological processes.

PBS-degradation deficient mutants (Mut-*nblA* and Mut-*nblB*) appeared greener than the wild-type strain under N-deficient conditions, which may be superficial. When excessive carbon was added to the nitrogen-limited cultures, growth was inhibited in the mutant strains, and they displayed non-bleaching cell damage (Figs. 4, 6). This non-bleaching cell damage might due the toxicity caused by excess active carbon without a corresponding increase in nitrogen, similar to the bleaching effect caused by high urea concentrations (Sakamoto et al. 1998). This highlights the additional role of PBS degradation in coping with high C/N stress environments. Previous studies primarily focused on the role of PBS degradation in nitrogen supplementation and photoprotection (Kiyota et al. 2014; Klotz et al. 2016; Nagarajan et al. 2019). Our work, however, reveals that in low-N or N-deficient cultures, growth inhibition of the mutant strains usually appeared under high but not low carbon conditions, indicating that PBS degradation is needed for adapting to high C/N stress environments. This has ecological implications. Especially considering increasing atmospheric CO_2_ emissions, cyanobacterial species capable of PBS degradation such as *Synechococcus,* may be better suited to nitrogen deficiency than those without this capability, such as *Prochlorococcus* (Ulloa et al. 2021). Further physiological and ecological studies are needed to support this hypothesis.

While the wild-type strain displayed a higher total C/N ratio under high C/N stress compared to the PBS-degradation deficient mutants (Mut-*nblA* and Mut-*nblB*) (Fig. 5C), this does not necessarily indicate a more severe active C/N imbalance in the wild-type. Conversely, the true active C/N ratio within the mutant strains might be higher than that of the wild-type strain. The large amount of nitrogen stored in PBS of the wild-type can be readily used for de novo synthesis essential amino acid and other cellular processes. However, in the PBS-degradation deficient mutants, this nitrogen source is unavailable. Therefore, the mutants are subjected to greater nitrogen deficiency stress, and their intracellular 2-OG content is higher than that of the wild-type strain (Fig. 6D). Consequently, the excess carbon in these mutants cannot be effectively used. This is further compounded by their inability to store carbon efficiently due to protein synthesis inhibition and the lack of glycogen and PHB synthesis. TEM images also suggested that the PBS-degradation deficient mutants accumulated less glycogen and PHB compared to the wild-type (Fig. 6C, E). The presence of excess, unusable carbon within the mutants may have detrimental physiological effects. Transcriptome analysis support this notion, revealing downregulation of CCM genes in the mutants (Fig. 7). This downregulation suggests a mechanism to reduce further carbon uptake and potentially mitigate the imbalance. Additionally, the mutant strains upregulated pathways related to nucleotide metabolism and amino acid synthesis, likely in an attempt to use the limited available nitrogen more efficiently and maintain an active C/N balance (Fig. 7). However, these compensatory strategies appear to be less effective than wild-type’s ability to degrade PBS for C/N homeostasis. In addition, the expression of genes involved in photosynthetic apparatus assembly pathway was generally down-regulated (Fig. 7), suggesting that high C/N conditions also affected photosynthesis in mutants. The transcription of *psbA* gene was up-regulated (Fig. 7; Supplementary Table S1). The D1 protein encoded by *psbA* is a major target of light-mediated PSII damage. To overcome photodamage of PSII, the damaged D1 protein was degraded and replaced by newly synthesized protein (Dai et al. 2014; Komenda et al. 2012). Based on this mechanism, we speculated that the upregulated expression of the D1 protein in the mutant strain under high C/N ratio conditions may be due to the induction of photodamage to PSII caused by the toxicity of C/N imbalance. However, the underlying mechanism requires further investigation. Ultimately, the high C/N environments lead to non-bleaching cell damage or death in the mutant strains, which highlighted the role of PBS degradation in maintaining C/N homeostasis and cellular health.

Previous studies indicate that when nitrogen is deprived, metabolic flux switches from pathways leading to amino acid synthesis towards glycogen accumulation, which serves as sink for newly fixed CO_2_ during N-deficiency (Forchhammer et al. 2019). There is a linear relationship between PBS degradation and glycogen synthesis (Damrow et al. 2016; Gründel et al. 2012). In *Synechocystis* 6803, mutants with glycogen synthesis defect were also unable to degrade PBS and respond appropriately to nitrogen deficiency (Forchhammer and Schwarz 2019; Gründel et al. 2012; Jackson et al. 2015; Kiyota et al. 2014). These observations, together with our results, suggest that PBS degradation is one of several strategies for cyanobacteria use to adapt to C/N imbalances. Both nitrogen acquisition through PBS degradation and heterocyst differentiation, as well as carbon consumption and storage mediated by the accumulation of polycarbon compounds such as PHB and glycogen, are strategies for maintaining C/N balance. Collectively, these observations suggest that future studies should focus on a more comprehensive understanding of metabolic processes at both ends of the C/N spectrum, rather than solely on nitrogen acquisition.

**Fig. 8:**
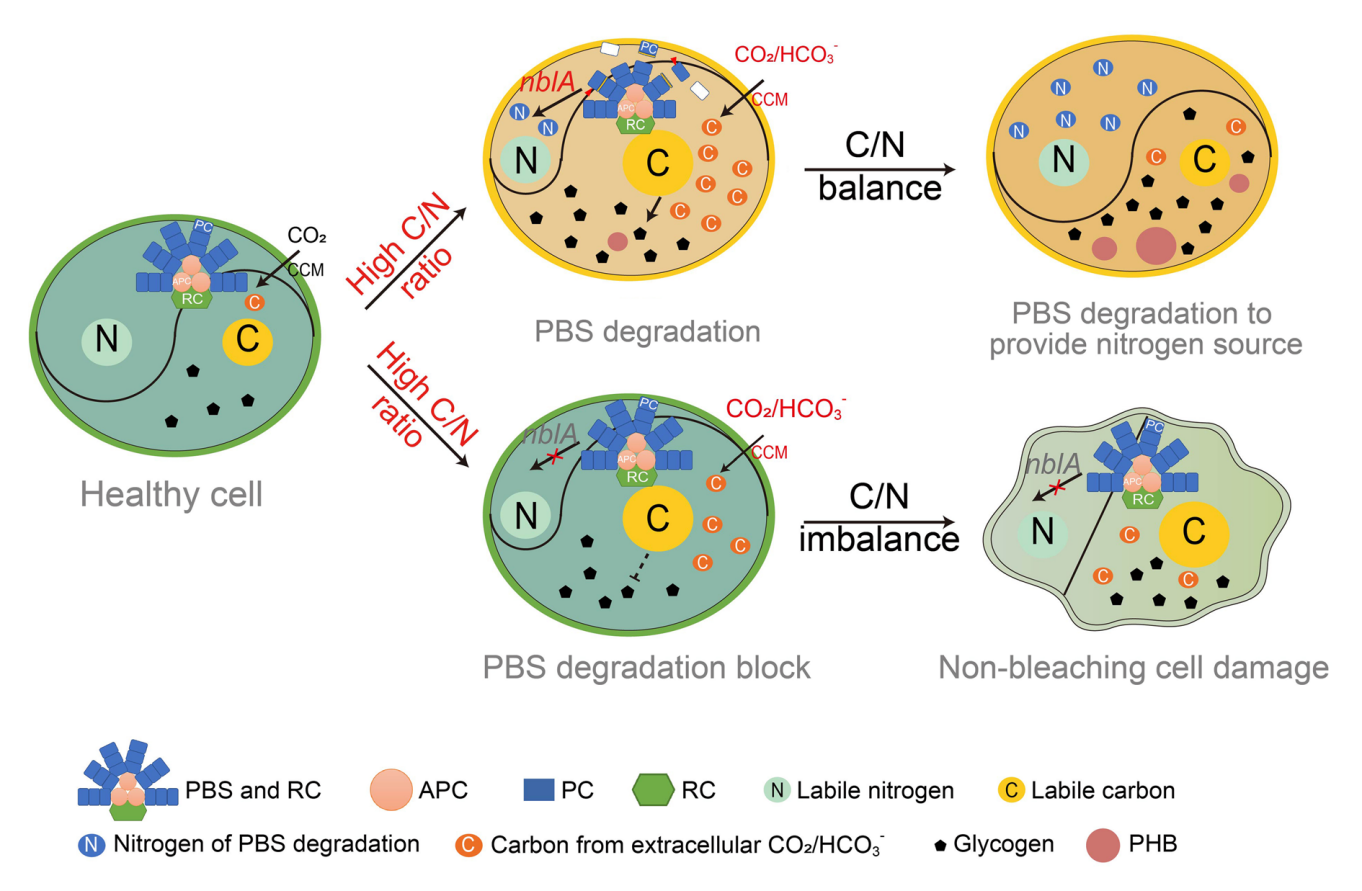
Model for PBS degradation maintains C/N balance in cyanobacteria. When cultured under high C/N ratio conditions, cyanobacteria maintain C/N balance by degrading PBS, accumulating PHB and glycogen and downregulating CCM-related genes. PBS-degradation deficient mutants cannot use nitrogen in PBS, and their ability to synthesize glycogen and PHB is inhibited. Therefore, even if the mutants also downregulate CCM, it still cannot maintain C/N balance. *RC*, photosynthetic reaction center; *PC*, phycocyanin; *APC*, allophycocyanin; *PHB*, polyhydroxybutyrate

Our findings support the existing model as shown in Fig. 8, where PBS degradation plays a role in maintaining C/N balance within cyanobacteria. When the C/N balance is disrupted, degradation of PBS contributes up to one-third of total protein turnover and releases nitrogen for essential processes like amino acid synthesis and cell growth (Kiyota et al. 2014). Additionally, excess carbon be rapidly stored in the form of glycogen and PHB within the cell matrix, further maintaining the bioavailable C/N balance. This explains why the total C/N ratio of the wild-type cells increased under high C/N conditions, but the readily bioavailable carbon and nitrogen remained balanced through PBS degradation and carbon storage. In contrast, PBS-deficient mutants (Mut-*nblA* and Mut-*nblB)* lacked the ability to use nitrogen from PBS and store excess carbon as PHB and glycogen. This dual impairment resulted in growth inhibition, cell damage, and cell death. In summary, our research emphasizes the role of PBS degradation in maintaining C/N balance under high C/N stress. It functions as a two-pronged strategy, providing nitrogen and facilitating carbon management. More generally, our work highlights the importance of studying both sides of the C/N spectrum, not just nitrogen acquisition, in future research on cyanobacterial PBS degradation.

## Supplementary Information

Below is the link to the electronic supplementary material.

Supplementary file1 (DOCX 2494 KB)

Supplementary file2 (ZIP 17 KB)

## Data Availability

Data supporting this work are included in this published article and its supporting information files. Sequence data from this paper have been submitted to the NCBI databases under accession number PRJNA1099755. Address is as follows: https://www.ncbi.nlm.nih.gov/bioproject/1099755.
