# Supplementary material for: Physiological and molecular evidence for phycobilisome degradation in maintaining carbon and nitrogen balance of cyanobacteria
Source: Mar Life Sci Technol. 2025 Apr 25;7(2):218–30. doi: 10.1007/s42995-025-00290-0 (PMC12102436; doi:10.1007/s42995-025-00290-0)
Supplement: Supplementary file 1 — Supplementary file1 (DOCX 2494 KB) [file 42995_2025_290_MOESM1_ESM.docx]

**Supplementary information**

**Fig. S1**
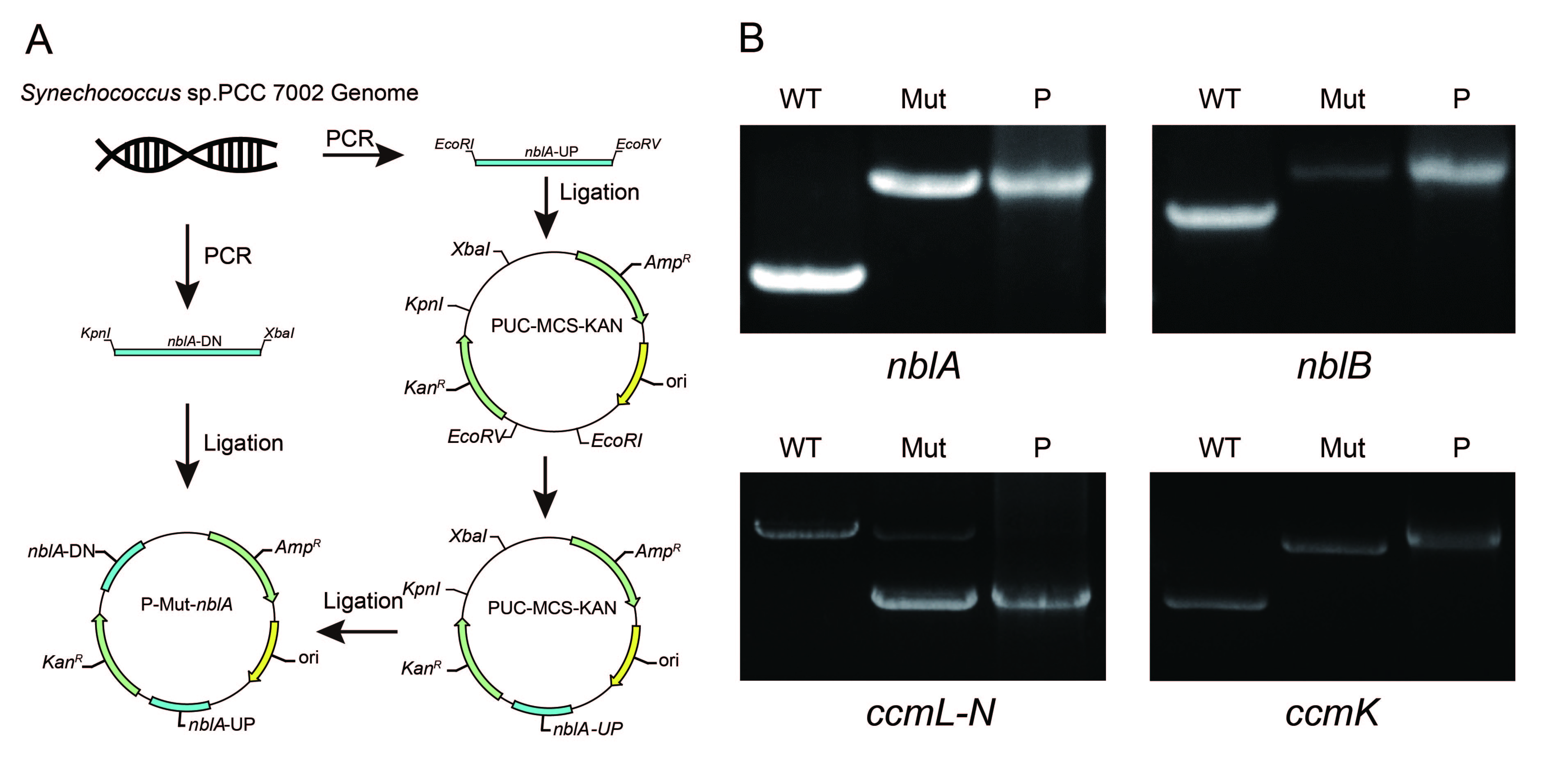


**Fig S1.** The construction of knockout plasmids, and PCR detection of the strains of *Synechococcus* 7002. A Schematic diagram of construction of plasmid for knockout strains (taking Mut-*nblA* as an example). B The PCR detection of the coresponding mutants of *Synechococcus* 7002. In each photo, from left to right is the results of PCR with the genome of *Synechococcus* 7002 (WT), the mutants (Mut), and the knockout plasmids (P), respectively.

**Fig. S2**


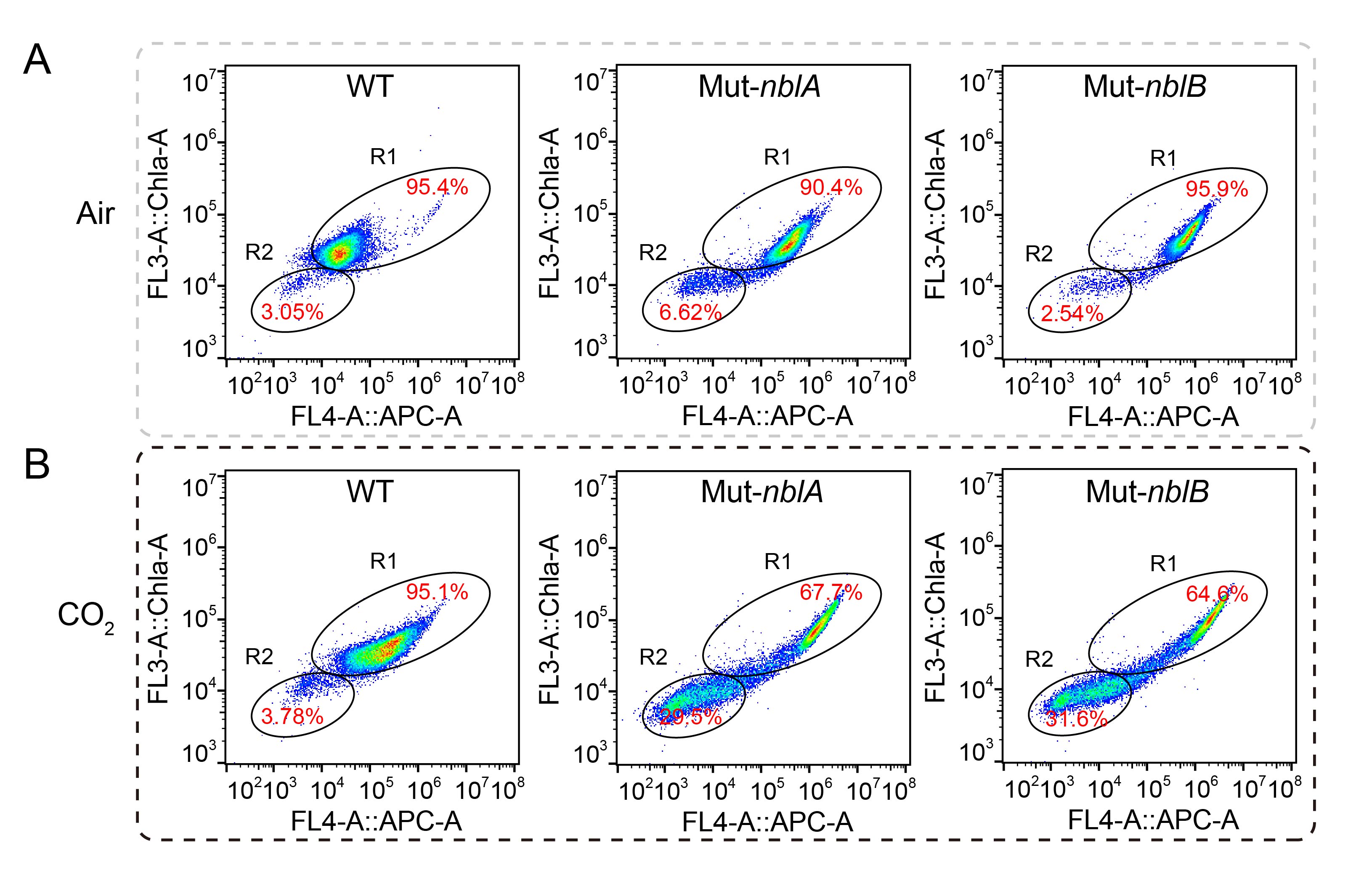


**Fig S2.** Flow cytometry scatterplots of the wild-type strain, Mut-*nblA* and Mut-*nblB* strains under low-N with air (A) or 2000 ppm CO_2_ (B) conditions for 4 days. The x-axis is APC auto-fluorescence of cells, the y-axis is Chl *a* auto-fluorescence of cells.

**Fig. S3**
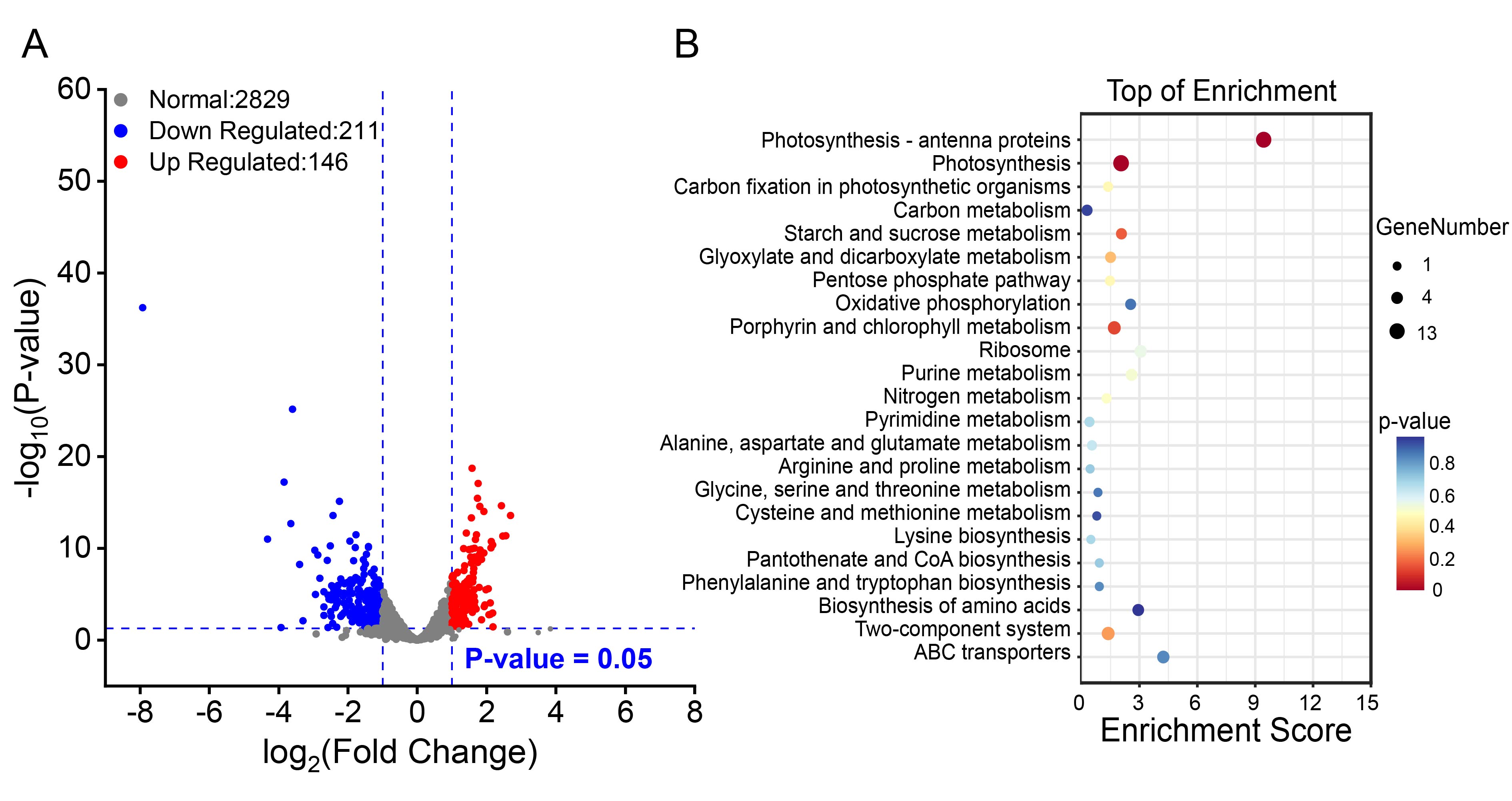


**Fig S3.** Differentially expressed genes (DEGs) analysis of Mut-*nblA* strain compared to wild-type strain of *Synechococcus* 7002 under low-N conditions with addition of 7.7 mM NaHCO_3_. A DEGs of Mut-*nblA* strain compared with wild-type strain. Splashes were for different genes, among which grey dots were genes with no significant discrepancy, red dots were genes significantly up-regulated and green dots were significantly down-regulated. B KEGG pathway enrichment analysis of DEGs.
